# Hepatoprotective Effects of a Chinese Herbal Formula, Longyin Decoction, on Carbon-Tetrachloride-Induced Liver Injury in Chickens

**DOI:** 10.1155/2013/392743

**Published:** 2013-02-21

**Authors:** Chunguang Wang, Tie Zhang, Xuemei Cui, Shuang Li, Xinghua Zhao, Xiuhui Zhong

**Affiliations:** College of Traditional Chinese Veterinary Medicine, Agricultural University of Hebei, Baoding 071001, China

## Abstract

The objective of this study is to establish poultry liver injury model induced by (CCl_4_) and seek effective hepatoprotective herbals for clinical application. Different doses of CCl_4_ dissolved in vegetable oil (1 : 1, *V/V*) were injected via pectoral muscle to induce acute liver injury model in chickens. An herbal formula, Longyin decoction, was prepared for hepatoprotection test on chicken acute liver injury models. The pathologic changes of the liver were observed, and the activities of ALT and AST were, respectively, detected to evaluate the hepatoprotective effects of Longyin decoction on chickens. The chicken acute liver injury model was successfully established by injecting CCl_4_ via pectoral muscle. The best dose of CCl_4_ inducing chicken liver injury was 4.0 mL/kg*·*BW (body weight). The results of qualitative determination by HPTLC showed that the components of Longyin decoction contained *Gentian, Capillaries, Gardenia,* and *Bupleurum root*. In the high-dose Longyin group and the middle-dose Longyin group, the pathological changes of the damaged liver were mitigated and the activities of ALT and AST in serum were reduced significantly. Longyin decoction has obvious hepatoprotective effect on acute liver injury induced by CCl_4_.

## 1. Introduction 

The liver is the largest substantive gland and an important metabolic organ. It has many important and complex physiological functions, including metabolism, excretion, detoxification, and generating a variety of coagulation factors. With the expanding of intensive farming industry, the disease factor on poultry hazards has become increasingly serious. Poultry liver injury is a clinically common disease. There are many disease causes including nutritional factors, disease factors, parasite factors, and poisoning factors [1]. The main and basic pathologic symptom of many liver diseases is swelling, degeneration, necrosis, and apoptosis of hepatic cells [2]. The poultry liver injury can lead to low performance and even death and has caused a great economic loss in poultry industry. At present, due to the lack of effective hepatoprotective drug in clinic, strengthening the research and development of hepatoprotective drugs has important significance to improve animal health. Chinese herbal medicine has many advantages like little side-effect, low cost, and little drug resistance. Thus, Chinese herbal preparations have been widely used in the animal health and disease prevention and control. 

The CCl_4_ can produce high toxicity in the metabolic process, and thus the vegetable oil solution of CCl_4_ is commonly used in the establishment of liver injury model [3]. The main acute toxic effects of CCl_4_ on animal body mainly manifest in inhibiting the central nervous system and damaging the kidney, liver, and lung [4]. The subacute toxicity of CCl_4_ mainly shows a dose-dependent elevation of the various enzyme levels in serum with the amount of CCl_4_ [5].

The mice and other mammals are often used as experimental animals to establish liver injury model in the field of human medicine and veterinary medicine [6–8]. With the rapid development of the poultry industry and taking into account the physiological characteristics of poultry, it is of urgent necessity to establish a CCl_4_-induced liver damage model of the target poultry. This model will reflect more accurately the changes of metabolism, function, and histological structures in poultry body.

We established the chicken liver injury model induced by CCl_4_ to provide basis for practical application of poultry pathology and clinical diagnosis. At the same time we also studied the hepatoprotective effects of Longyin decoction. The best dose of CCl_4_ and the possible hepatoprotective mechanism of the herbals were investigated in order to provide theoretical basis for research and development of hepatoprotective herbals.

## 2. Materials and Methods 

### 2.1. Chickens

A total of 360 ISA Brown layers at the age of 1 d were purchased from Shijiazhuang Jinyu Grandfather Chicken Farm. They were fed for 15 d according to the feeding standard of chicken (NY/T33-2004) promulgated by the Ministry of Agriculture of the People's Republic of China. Then 60 ISA Brown layers at the age of 15 d were selected to establish liver injury model according to the standards of health and body weight. The other 300 ISA Brown layers were used for hepatoprotecting study of Chinese herbal medicine. All procedures concerning animal treatments and experimentations in this study were reviewed and approved by the Institutional Committee for Ethical Use of Experimental Animals at Hebei Provincial Department of Animal Science.

### 2.2. Reagents

Longdan Xiegan powder was purchased from the Henan Shangdu Pharmaceutics Co., Ltd. This herbal product was used as a positive control drug, commercially available and documented in the Chinese Pharmacopoeia of Veterinary Drugs.

Reference herbs and reference herbal components were purchased from China Institute of Veterinary Drug Control. Silica gel G thin plate was purchased from Yantai Chemical Industry Research Institute. Carbon tetrachloride (CCl_4_) was purchased from the Beijing Chemical Reagent Company. 

The formula of Longyin decoction includes* Capillaries*, *Gentian, Gardenia*,* Bupleurum root, *and *Licorice*, where the five herbs are of equivalent weight (200 g). They were soaked in 10 times weight of distilled water for 30 min and then brought to boiling. After simmered for 20 min, they were filtered with four layers of gauze and the herbal soup was collected. Five times weight of distilled water was added into the remaining herbals for a second decoction for another 20 min and then filtered. The herbal soup collected at two times was put together and concentrated on a slow fire until the crude herbal content is l g/mL. Finally, the prepared Longyin decoction was sterilized and stored in refrigerator for use. 

### 2.3. Establishment of Chicken Acute Liver Injury Model by Injecting CCl_4_

A total of 60 healthy chickens were randomly divided into six groups, including five model groups (groups I–V) and blank control group, 10 chickens of each group. On the first and third days, the chickens were, respectively, challenged with the CCl_4_ peanut oil solution at the volume ratio of 1 : 1. The injection dose of the CCl_4_ peanut oil solution in groups I, II, III, IV, and V were 1.0, 2.0, 4.0, 8.0, and 16.0 mL/kg*·*BW, respectively. In the blank control group, the chickens were injected with peanut oil (4.0 mL/kg*·*BW) via pectoral muscle. During the whole trial, the clinical symptoms of these chickens in each group were monitored and recorded. On days 4 and 7, five chickens were selected from each group and the blood samples were collected via wing vein, and then the serum was separated. The activities of the ALT and AST in serum were detected by the automatic biochemical analyzer. The experimental data were analyzed with SPSS 11.5 statistical software. Moreover, the pathological changes of liver and other major organs were observed. The best dose of CCl_4_ was determined according to the minimum dose of CCl_4_ which can induce the visible necrotic lesions and can significantly increase serum aminotransferase activity in liver.

### 2.4. Detection of Longyin Herbal Formula by HPTLC

Four tests for the identification of *Gentian*,* Capillaries*, *Gardenia*, and *Bupleurum root* was carried out, three sample solutions (including sample solutions 1, 2, and 3) in each test. Preparation of sample solution was as follows: 10.0 mL Longyin decoction was evaporated to dryness with water bath and the residue was dissolved in 1.0 mL methanol. Moreover, in the preparation of sample solution for identification of* Bupleurum root*, the 10.0 mL Longyin decoction was firstly extracted with n-butanol, and then it was evaporated to dryness and dissolved in 1.0 mL methanol. Preparation of control drug solution was as follows: each of 1.0 g reference drug substance (*Gentian*,* Capillaries,* or *Gardenia*) was boiled and filtered; the filtrate was evaporated to dryness and then added 2.0 mL methanol. The reference drug substance *Bupleurum root* was firstly extracted with n-butanol, and then it was boiled, filtered and evaporated; finally, it was dissolved in methanol. Each of 1.0 mg gentiopicroside, geniposide, and saikosaponin-*α* was respectively, dissolved in 1.0 mL methanol and they were used as control solution. The negative Longyin decoction sample free of the corresponding drug was prepared according to the prescription. The preparation method of negative control solution was the same as that of the above sample solution. Each of 2.0–2.5 *μ*L prepared solution was used for identification of *Gentian*,* Capillaries*, *Gardenia, *and* Bupleurum root *by HPTLC method. 

### 2.5. Protection Test of Longyin Decoction on Liver

A total of 300 chickens were randomly divided into four treatment groups including high-dose Longyin group, middle-dose Longyin group, low-dose Longyin group, and drug control group. The two control groups include model control group and blank control group, 50 chickens of each group. The best dose of CCl_4_ to induce liver injury was determined according to the liver injury model test. Except the blank control group, the chickens in other groups were injected twice via pectoral muscle with CCl_4_ peanut oil solution (4.0 mL/kg*·*BW) on days 1 and 3. After the last injection (day 3), the chickens in each group were treated with different drugs for 5 days consecutively (from days 4 to 8). The chickens in the low-dose Longyin group, middle-dose Longyin group, and high-dose Longyin group were treated with different doses of the Longyin decoction (1.0, 2.0, and 4.0 mL/L, resp.) via drinking water. Chickens in the drug control group were treated with Longdan Xiegan powder (2.5 g/kg) mixed with feed. Those chickens in the model group and blank control group were not treated with herbals. On the first day after 5 days, herbal treatment (day 9), the blood samples were collected from the chickens in each group via wing vein, and the activities of ALT and AST in serum were detected. After blood sampling, the livers were picked and the lesions were observed. The liver tissues were sliced and stained with HE. The liver histological changes were observed and photographed with the OLYMPUS microscope, and the experimental data were analyzed with SPSS 11.5 statistical software. 

## 3. Results

### 3.1. Chicken Acute Liver Injury Model Induced by CCl_4_

 In the blank control group, the chickens kept good spirit and appetite and did not show any clinical symptoms. After first challenge with CCl_4_, the chickens in model groups showed slightly decreased reactivity and had no other obvious clinical symptoms. After second challenge, most of the chickens in model groups showed listlessness and decreased feed intake. In groups III, IV, and V, some chickens discharged yellowish green loose stools and showed standing instability and even recumbency. The appetite was recovered on day 6. 

**Figure 1:**
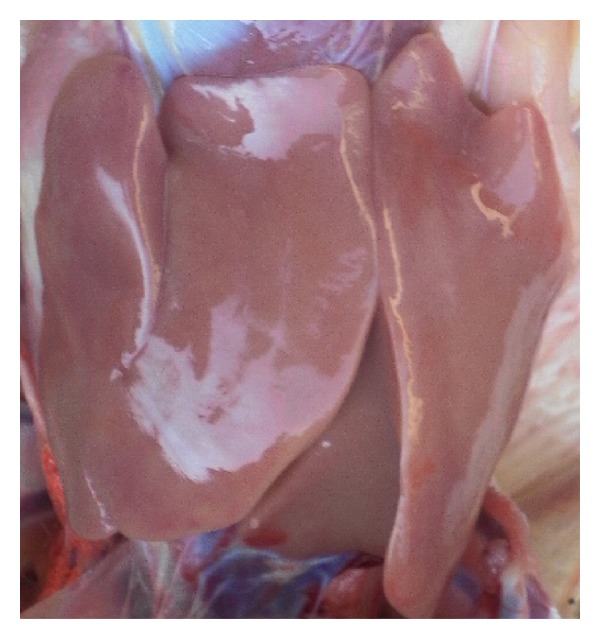
Chicken liver in group I. The liver is slightly larger and yellow in color.

**Figure 2:**
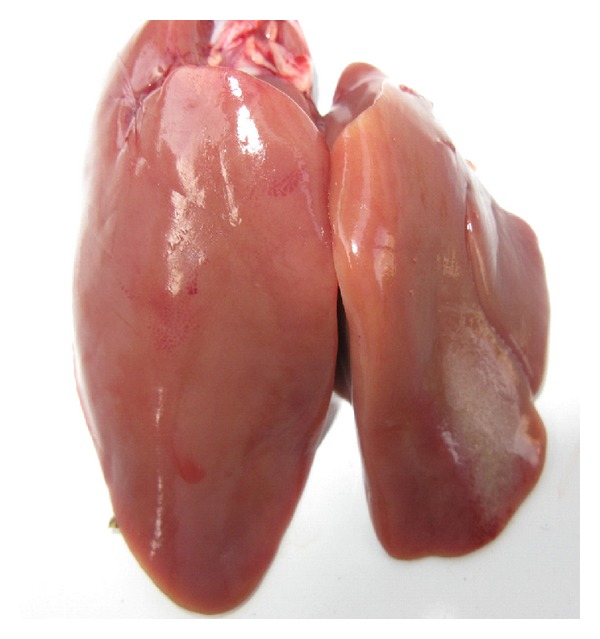
Chicken liver in group II. The superficial necrosis is found in liver edge.

**Figure 3:**
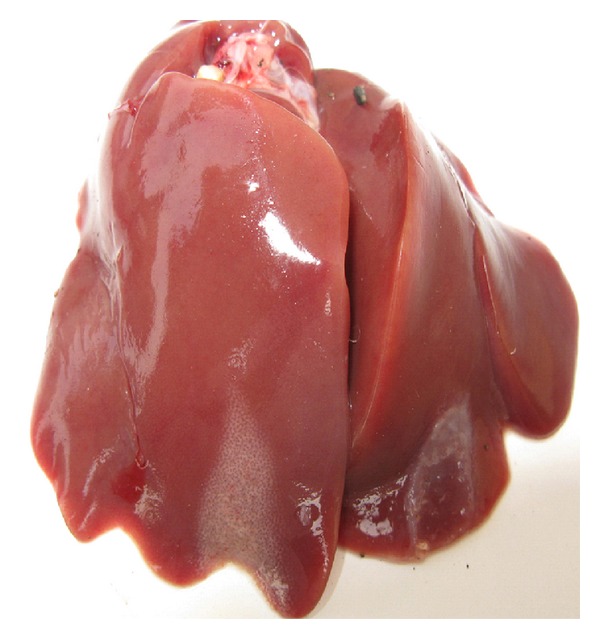
Chicken liver in group III. The multiple needle tip necrosis appears in liver.

**Figure 4:**
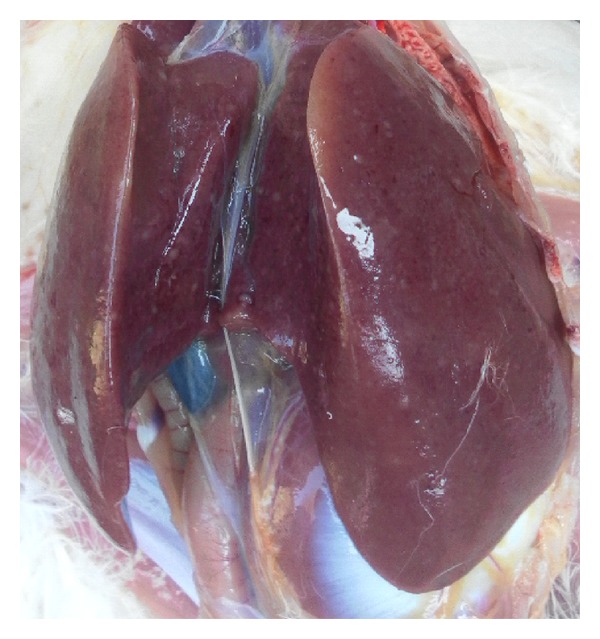
Chicken liver in group IV. The liver is larger and harder and shows large necrotic plaque.

**Figure 5:**
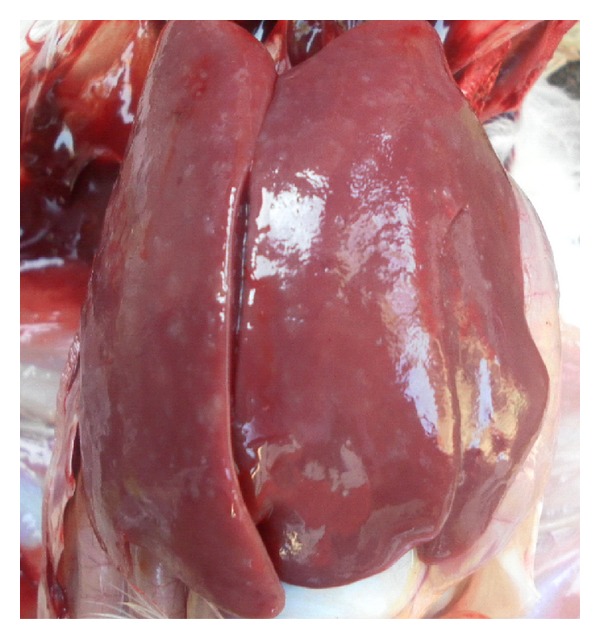
Chicken liver in group V. The liver is larger and harder and shows large necrotic plaque.

**Figure 6:**
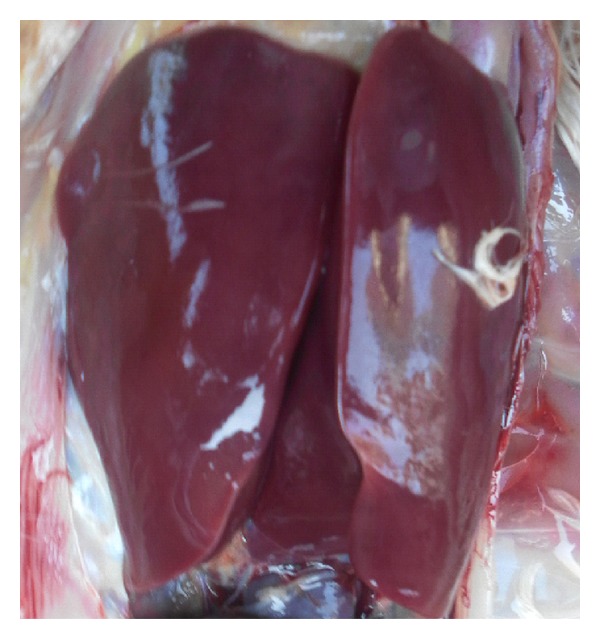
Chicken liver in blank control group. The liver is red, shiny, wet, flexible, and keeping normal volume.

Pathological changes of the liver were observed through clinical autopsy. In group I, the liver was slightly larger and showed slightly yellow color (Figure 1). In group II, the superficial necrosis was found in some chicken liver edge (Figure 2). In group III, the multiple needle tip necrosis appeared in chicken liver, the cecal tonsils had scattered bleeding points, and a small amount of bleeding point was observed at injection site and on endocardium or epicardium (Figure 3). In groups IV and V, much more liver lesions were noticed. The liver was significantly larger and harder, and showed large necrotic plaque. The liver edge was blunt thick and khaki. Moreover, the splenomegaly was larger and appeared piebald (Figures 4 and 5). In the blank control group, the liver was dark red, shiny, wet, flexible, and kept normal size (Figure 6).

 The activities of ALT and AST in serum were detected. The results showed that the activities of ALT and AST in serum were increased with the increased dose of CCl_4_. The changes of ALT were earlier and greater than that of AST. After challenge, both ALT activity on day 4 and AST activity on day 7 had extremely significant difference between group III and blank control group (*P* < .01). These indicated that injecting CCl_4_ peanut oil solution (4.0 mL/kg*·*BW) via pectoral muscle could cause increasing of ALT and AST activities in serum. 

According to the observation results of clinical symptoms, clinical autopsy, and aminotransferase activity detection, the best dose of CCl_4_ peanut oil solution inducing chicken acute liver injury was determined and was 4.0 mL/kg*·*BW (Table 1). 

### 3.2. Qualitative Determination of Longyin Decoction

**Figure 7:**
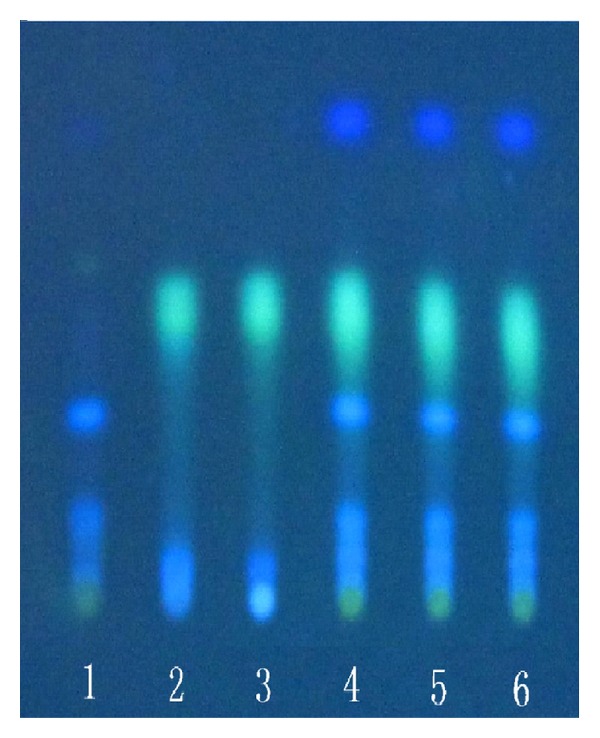
Thin-layer chromatogram of *Gentian* in Longyin decoction. 1: negative control solution free *Gnetian*; 2: control solution of gentiopicroside; 3: reference drug substance of* Gentian*; 4–6: sample solutions 1, 2, and 3.

**Figure 8:**
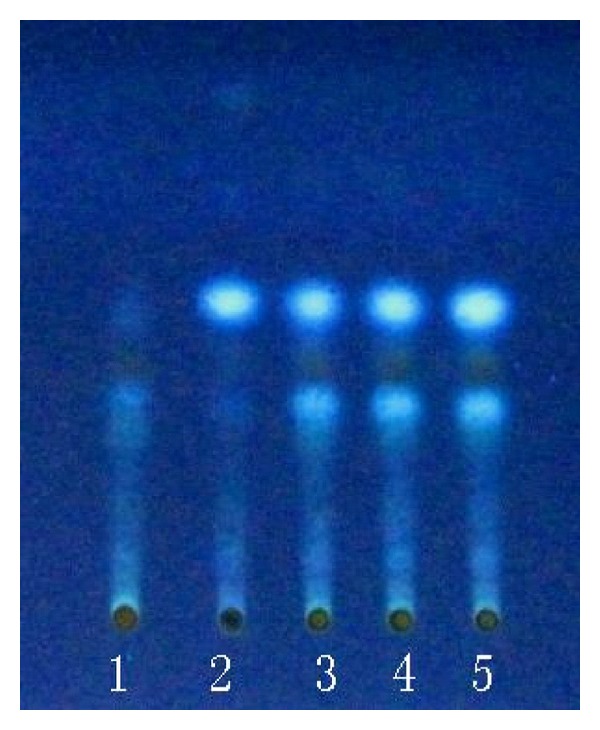
Thin-layer chromatogram of *Capillaries* in Longyin decoction. 1: negative control solution free *Capillaries*; 2: reference drug substance of *Capillaries*; 3–5: sample solution 1, 2, and 3.

**Figure 9:**
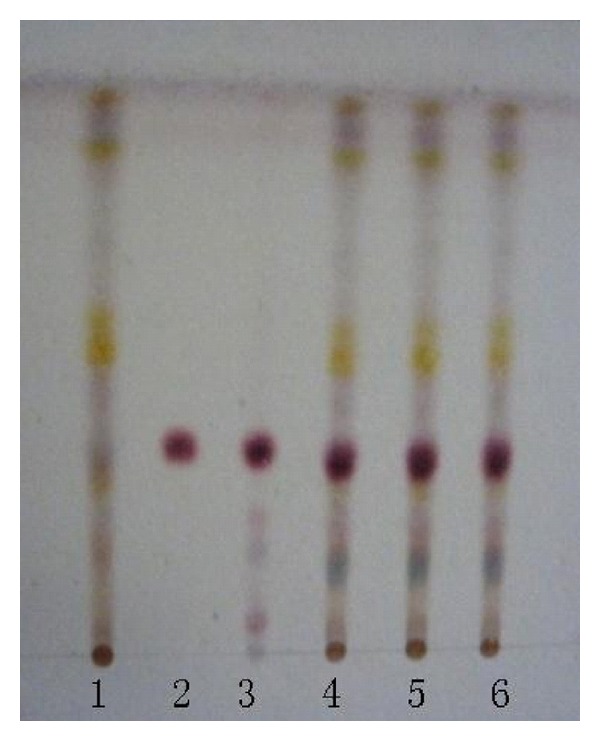
Thin-layer chromatogram of *Gardenia* in Longyin decoction. 1: negative control solution free *Gardenia*; 2: Control solution of geniposide; 3: reference drug substance of* Gardenia*; 4–6: sample solution 1, 2, and 3.

**Figure 10:**
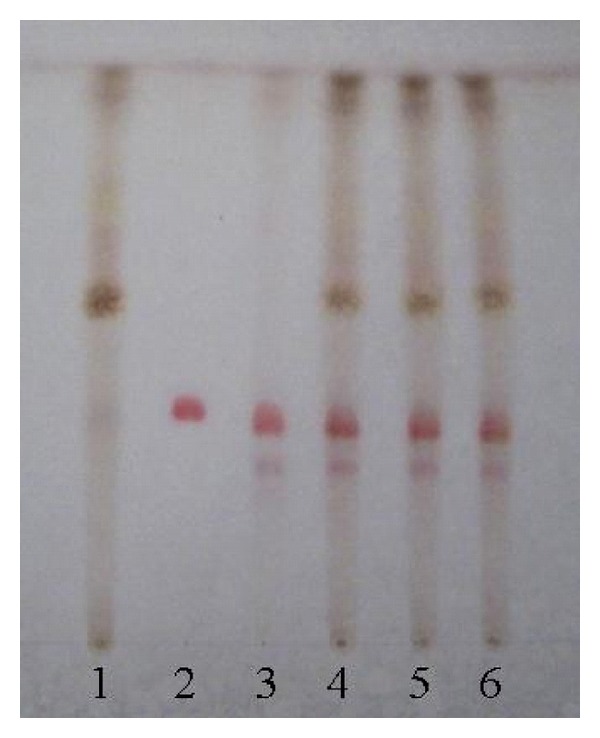
Thin-layer chromatogram of *Bupleurum root* in Longyin decoction. 1: negative control solution free *Bupleurum root*; 2: control solution of saikosaponin-*α*; 3: reference drug substance of* Bupleurum root*; 4–6: sample solution 1, 2, and 3.

 In chromatogram of sample solution, the same color spots were showed in corresponding position with that of control drug solution and control solution. In chromatogram of negative solution, there was no spot found in corresponding position (Figures 7, 8, 9, and 10). 

### 3.3. Protection Test of Chinese Herbal Medicine on Chicken Acute Liver Injury

The activities of ALT and AST in each treatment group were lower than that in the model control group with extremely significant differences (*P* < .01) (Table 2). The activity of ALT in serum had no significant difference between high-dose Longyin group, middle-dose Longyin group, and blank control group (*P* > .05). The ALT levels in middle-dose Longyin group and low-dose Longyin group were significantly different from that in drug control group (*P* < .05). In the low-dose Longyin group and drug control group, the ALT levels have extremely significant differences from that in blank control group (*P* < .01).

 The activity of AST in serum in each treatment group was significantly different from that in blank control group (*P* < .01). There was no significant difference of AST levels between high-dose Longyin group and middle-dose Longyin group (*P* > .05). The AST level of middle-dose Longyin group was significantly different from that of low-dose Longyin group (*P* < .05). 

At necropsy the color and volume of liver in blank control group were normal. In the model control group, the liver showed multiple needle tip necrosis. In the high-dose Longyin group, a few chickens showed slightly yellow at edge of liver. In the middle-dose Longyin group, low-dose Longyin group, and the drug control group, a small amount of needle tip necrosis appeared in chicken livers.

**Figure 11:**
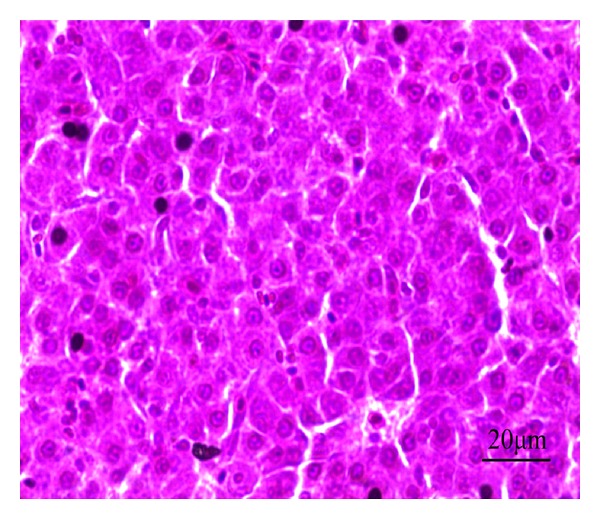
HE staining of chicken liver slice in blank control group. The hepatic lobule has complete structure and the liver cell has regular shape, 400x.

 The liver histopathology of chickens in each group was examined. In the blank control group, the hepatic lobules centered around the central vein, the hepatocyte tubes being surrounded by liver cells, showed radially walk to the lobule edge. There was a small amount of red blood cells and white blood cells in the liver sinusoids between the hepatocyte tubes. The liver cells were cube with plump cytoplasm and normal structure, and the nucleus was in regular shape and located in the center of the cell (Figure 11). 

**Figure 12:**
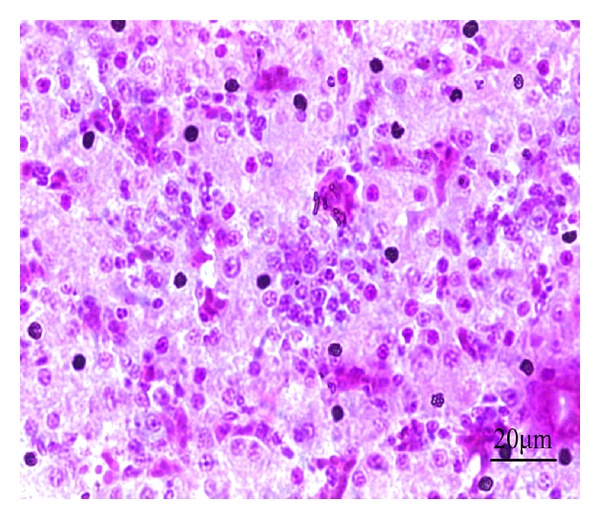
HE staining of chicken liver slice in model control group. The hepatocyte tubes arranged haphazardly with severe congestion and inflammatory cell infiltration, liver cells appear necrosis with nuclear fragmentation, 400x.

In the model control group, the hepatocyte tubes were arranged haphazardly with severe congestion and inflammatory cell infiltration. Liver cells had irregular contour with many vacuoles formed by fatty degeneration. Some areas of liver showed necrosis, cell rupture, nuclear condensation, fragmentation, and aggregation (Figure 12).

**Figure 13:**
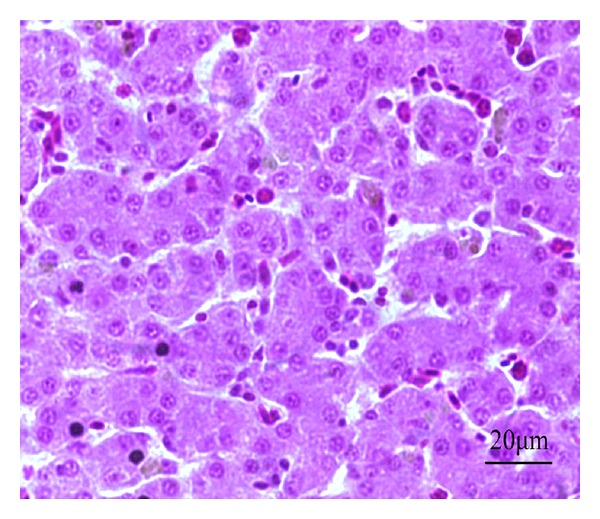
HE staining of chicken liver slice in high-dose Longyin group. The hepatic lobules have clear structure, the hepatocyte tubes arranged orderly and closely, 400x.

**Figure 14:**
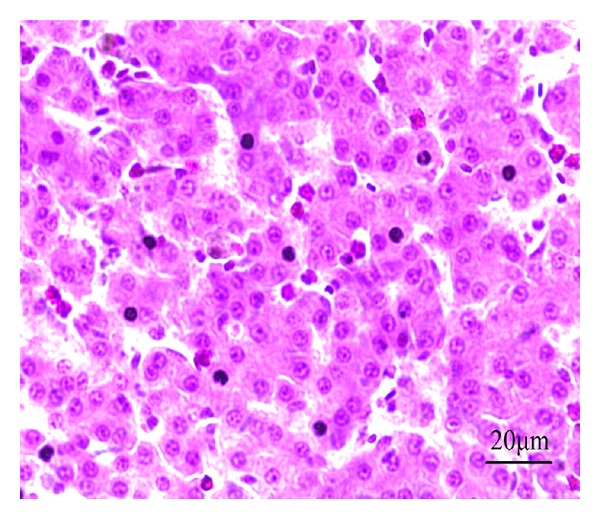
HE staining of chicken liver slice in middle-dose Longyin group. The hepatocyte tubes arranged orderly but slightly loose with mild congestion, the cytoplasm is reduced, 400x.

**Figure 15:**
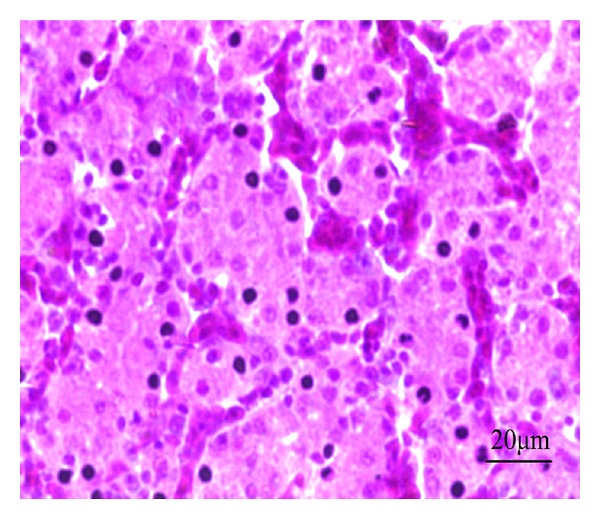
HE staining of chicken liver slice in low-dose Longyin group. The hepatocyte tubes arranged in disorder with congestion and inflammatory cell infiltration, and the liver cells show pale watery cytoplasm, 400x.

In the high-dose Longyin group, the hepatic lobules had clear structure, the hepatocyte tubes were arranged orderly and closely, and the liver sinusoids showed the mild congestion (Figure 13). In the middle-dose Longyin group, the hepatic lobule structure was normal, the hepatocyte tubes arranged orderly but slightly loose with mild congestion, and the cytoplasm was reduced (Figure 14). In the low-dose Longyin group, the the hepatocyte tubes were arranged in disorder with congestion and inflammatory cell infiltration, and the liver cells showed pale watery cytoplasm (Figure 15).

**Figure 16:**
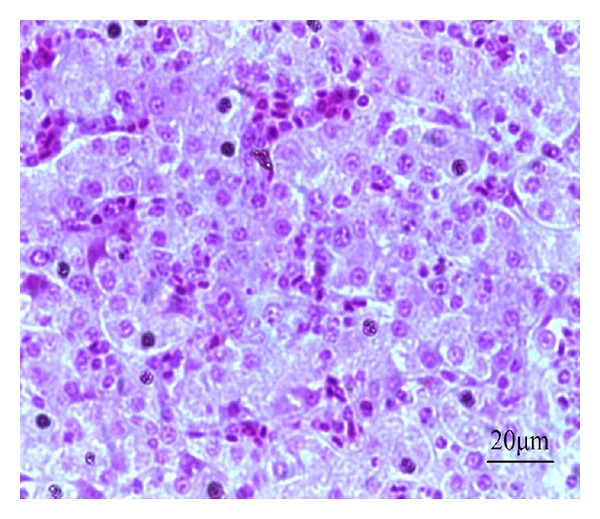
HE staining of chicken liver slice in drug control group. The liver shows congestion and inflammatory cell infiltration, and the liver cell shows granular degeneration, 400x.

 In the drug control group, the grade of liver lesions was close to those in the low-dose Longyin group, we also found the congestion and inflammatory cell infiltration, as well as granular degeneration of the liver cells (Figure 16). 

## 4. Discussion

### 4.1. Damage Effects of CCl_4_ on Liver

 CCl_4_ is a strong proliver toxicant and can produce lively trichloromethyl radicals and chlorine-free radicals after getting into body via different pathways. It can cause the lipid peroxidation of endoplasmic reticulum membrane and cell membrane, and then damage the liver cell. In addition, the CCl_4_ can reduce the degeneration, necrosis, fibrosis, and canceration of liver [9]. Therefore, we can observe the inflammatory cell infiltration, fatty degeneration, cloudy swelling, ballooning degeneration, and liver necrosis in liver slices. The destruction of liver cell membrane structure and functional integrity causes the increase of the soluble enzymes (ALT and AST) activity. Then these soluble enzymes get into blood from the liver cells thus, causing the increase of serum enzyme activity. Therefore, the activities of ALT and AST in serum are a sensitive index of liver damage and can reflect the degree of liver damage and necrosis to a certain extent [10]. 

In this study, injecting CCl_4_ oil solution (>4.0 mL/kg*·*BW) via pectoral muscle can result in chicken liver substantial necrotic lesions and great increases of ALT and AST levels in serum. In early period of trial, the change speed of ALT is greater than that of AST. However, we had verified that the damage of CCl_4_ on chicken liver shows a toxic cumulative effect, namely, the chicken liver injury and increase of serum transaminase levels induced by CCl_4_ require certain duration of action and a higher challenge dose. It indicates that chicken has certain tolerability to CCl_4_ oil solution.

### 4.2. Protection Effects of Longyin decoction on Liver

According to the concept of traditional Chinese Veterinary medicine, liver damage is of *Qi* stagnation pattern. The pathogenesis is damp-heat stagnation resulting in liver *Qi* difficulties in moving, and the treatment principle should be to dispel damp-heat and smooth the liver [11]. A large number of clinical practices and experimental researches have confirmed that the Chinese herbal medicine has significant protective effects on liver injury and has unique advantages on removing jaundice and liver protection therapy. The treatment of Chinese herbal medicine on liver injury is mainly manifested in the inhibition of lipid peroxidation, stabilization of biological membrane, improving hepatic microcirculation, inhibiting liver cell apoptosis, and inflammatory cytokines [12]. Previous studies have reported many single Chinese herbs with hepatoprotective effects as well as their active ingredients, including *Scutellaria*, *Astragalus*, *Herba Rhodiola*, *Polygonum cuspidatum*, *Radix lithospermi*, *Angelica sinensis*, *Radix salviae miltiorrhizae*, Cordyceps polysaccharide, Polysaccharide of *Grifola Frondosa (PGF)*, Geranium flavonoids, Rhizoma Anemarrhenae total flavone, Momordica glycoside, tetramethylpyrazine (TMP), Fly thistle element, schizandrin, tetrandrine glycyrrhizin, and others [1, 13–20]. Luo et al. have verified that the skullcap and baicalin have protective effects on rat primary hepatocytes damaged by ethanol. Particularly, the optimal concentration *in vitro* of the baicalin is 0.05 *μ*g/mL [13]. The *PGF* can inhibit the liver cell apoptosis by regulating expression of apoptosis-related protein Bcl-2 and Bax. It also can reduce the activity of serum transaminase, increase the SOD content and decrease the MDA content to enhance the ability of scavenging free radicals [1].

In addition, a great number of studies have demonstrated that the protective effect of compound Chinese herbal medicine on liver is more comprehensive. The commonly used Chinese herbal prescription includes the modified Xiaochaihu Decoction, Longdan Xiegan powder, compound Caiyu decoction, Peony and Licorice decoction, Yinchenhao decoction, Glycyrrhizae decoction for purging stomach fire, and Sini decoction [21–27]. 

The Longdan Xiegan decoction can decrease the serum lactate dehydrogenase (LDH) and ALT levels of mice with liver injury and reduce the cell degeneration and liver necrosis induced by CCl_4_ [22]. The compound Caiyu decoction can effectively prevent the immunologic damage of mouse liver induced by BCG + lipopolysaccharide (LPS) and significantly decrease the AST and ALT levels in serum [23]. 

Many previous researches on hepatoprotective effect of traditional Chinese medicine focus on mammalian (mouse and rat), and research subjects which focuses on poultry has not been reported. 

The Longyin decoction was modified and prepared according to the classic prescription of traditional Chinese medicine “Oriental wormwood Decoction” in Waitai miyao written by Wang Tao of Tang Dynasty. And it includes *Capillaries*, *Gentian, Gardenia, Bupleurum root, *and *Licorice *[28]. *Gentian* which refers to the root and rhizome of *Gentiana manshurica Kitag.; Gentiana scabra Bge.; Gentiana triflora Pall., *or *Gentiana rigescens Franch. ex Hemsl*. *Capillaries *was whole herb or aboveground parts of *Artemisia scoparia Waldst. et Kit. *or* Artemisia capillaris Thumb. Gardenia *is the dried ripe fruit of* Gardenia jasminoides Elli*. *Bupleurum root* is the dried root of *Bupleurum chinense DC.* or *Bupleurum scorzonerifolium Willd*.* Licorice* refers to the dried root and rhizome of *Glycyrrhiza uralensis Fisch.*;* Glycyrrhiza inflate Bat. *or *Glycyrrhiza glabra L*. *Long dan* (*Gentian),* and *Yin chen (Capillaries)* functions to treat jaundice according to the Chinese herbal book, while *Zhi zi* (*Gardenia), chai hu (Bupleurum root)*, and *gancao* (*Licorice*) work together to assist *Long dan* and *Yin chen*. Take the first words of the two main herbs, hence the formula name is Longyin decoction. In the hepatoprotective test of Longyin decoction on liver, the serum transaminase activity and the liver microstructure in each experimental group were detected and observed, respectively. The results showed that the Longyin oral liquid could reduce liver necrosis and decrease serum transaminase activity (ALT and AST) and had protective effects on liver injury induced by CCl_4_. 

## Figures and Tables

**Table 1:** Detection of serum biochemical indicators after modeling test.

Groups	ALT (U/L)		AST (U/L)	
	On day 4	On day 7	On day 4	On day 7
Group I	2.21 ± 0.32	2.41 ± 0.42	207.64 ± 46.74	217.65 ± 37.84
Group II	2.30 ± 0.42	2.55 ± 0.54*	214.91 ± 41.09	233.66 ± 45.54*
Group III	3.22 ± 0.35**	3.75 ± 0.40**	232.41 ± 32.46	264.07 ± 31.29**
Group IV	3.54 ± 0.40**	4.22 ± 0.33**	248.08 ± 53.62*	284.64 ± 35.06**
Group V	3.88 ± 0.34**	4.83 ± 0.57**	265.22 ± 39.74**	298.07 ± 42.70**
Bland control group	2.11 ± 0.25	2.12 ± 0.35	198.55 ± 33.62	194.39 ± 25.16

Values are mean ± SE, *n* = 5.

*means significant difference (*P* < 0.05); **Means extremely significant difference (*P* < 0.01).

**Table 2:** Detection of serum biochemical indicators after treatment by Longyin decoction.

Groups	ALT (U/L)	AST (U/L)
Low-dose Longyin group	2.67 ± 0.53^BCb^	239.48 ± 47.96^Bb^
Middle-dose Longyin group	2.42 ± 0.59^BCDc^	221.24 ± 50.62^BCcd^
High-dose Longyin group	2.37 ± 0.67^CDc^	216.76 ± 49.01^Cd^
Drug control group	2.69 ± 0.76^Bb^	237.81 ± 38.32^BCbc^
Model control group	3.83 ± 0.46^Aa^	273.50 ± 36.65^Aa^
Blank control group	2.19 ± 0.58^Dc^	192.40 ± 30.92^De^

Values are mean ± SE, *n* = 50.

Different lowercase letters mean significant difference (*P* < 0.05); different majuscule letters mean extremely significant difference (*P* < 0.01).
